# Research Advances in the Synthesis and Regulation of Apple Anthocyanins

**DOI:** 10.3390/biology14101322

**Published:** 2025-09-25

**Authors:** Haidong Bu, Guangjun Gu, Yinghui Hu, Yue Yang, Ling Yang, Hui Yuan, Wenquan Yu

**Affiliations:** 1 Key Laboratory of Cold Region Fruit Breeding and Cultivation, Mudanjiang Branch of Heilongjiang Academy of Agricultural Sciences, Mudanjiang 157000, China; buhaidong11@126.com (H.B.);; 2 Research Institute of Pomology, Chinese Academy of Agricultural Sciences, Xingcheng 125100, China; 3 Key Laboratory of Fruit Postharvest Biology, College of Horticulture, Shenyang Agricultural University, Shenyang 110866, China

**Keywords:** apple, anthocyanin, biosynthesis, transcriptional regulation, environmental factors

## Abstract

Anthocyanins are key pigments responsible for apple fruit coloration, influencing not only its appearance and marketability but also contributing significantly to its nutritional and health benefits. In their natural state, anthocyanins are chemically unstable. However, glycosylation modifies them into anthocyanin derivatives known as anthocyanin glycosides, which exhibit markedly enhanced stability and improved water solubility. As a result, most naturally occurring anthocyanins exist in the form of anthocyanin glycosides. The biosynthesis of anthocyanins involves a series of structural genes within the phenylpropanoid and flavonoid pathways (including *PAL*, *C4H*, *4CL*, *CHS*, *CHI*, *F3H*, *DFR*, *ANS*/*LDOX*, and *UFGT*). The MYB–bHLH–WD40 transcriptional complex serves as the core regulatory mechanism controlling anthocyanin synthesis, with additional transcription factors also playing important roles. This review systematically summarizes recent advances in the structural characteristics, biosynthetic pathways, molecular regulatory mechanisms, and environmental factors affecting anthocyanin accumulation in apples. These insights are important both for consumer health and for breeding apple cultivars with enhanced anthocyanin content.

## Simple Summary

Significant advances have been made in the study of apple anthocyanins through molecular biology techniques. The anthocyanin biosynthetic pathway has been largely elucidated, and key regulatory factors such as the MYB–bHLH–WD40 transcription complex, along with other elements influencing anthocyanin synthesis, have been identified. This article highlights recent progress in understanding the synthesis and regulation of apple anthocyanins and summarizes the mechanisms by which environmental factors and phytohormones affect fruit coloration. The review aims to provide a comprehensive foundation for the efficient development of apple varieties with high anthocyanin content.

## 1. Introduction

Apple (*Malus* × *domestica*) is one of the most widely cultivated temperate fruits worldwide. The color of the fruit peel is a critical indicator of external quality and directly influences its market value [1,2]. Apple peel coloration is primarily determined by background and overcolor, with anthocyanins being the key pigments responsible for red overcolor [3,4]. In addition, red-fleshed apple varieties accumulate anthocyanins not only in the peel but also in the flesh, as exemplified by low-acid cultivars such as ‘Ruby Sweet’ and ’Rose Pearl’ [5]. Anthocyanins are flavonoid secondary metabolites widely distributed in plants. They impart vibrant colors to plant tissues. Additionally, they participate in various physiological processes, such as attracting pollinators and helping plants cope with both abiotic and biotic stresses [6,7,8,9]. For humans, anthocyanins exhibit remarkable antioxidant activity, with free radical scavenging capacities 20–50 times greater than those of vitamins C and E. They also offer potential health benefits, including visual protection, prevention of cardiovascular diseases, and anti-carcinogenic effects [10,11,12,13].

To date, more than 600 types of anthocyanins have been identified in nature [14]. The most common natural anthocyanidins include cyanidin, delphinidin, malvidin, petunidin, peonidin, and pelargonidin, among which cyanidin and its derivatives are the most prevalent [14,15]. Different anthocyanidins contribute to distinct colors in plant tissues; for instance, cyanidin derivatives typically appear red, delphinidin, malvidin, and petunidin derivatives tend to be blue-purple, peonidin derivatives are often magenta, and pelargonidin derivatives usually show orange-red hues [13]. Anthocyanidins are chemically unstable in their free form. Glycosylation leads to the formation of anthocyanins, which significantly enhances molecular stability and improves water solubility. Therefore, in nature, most anthocyanidins exist in the form of anthocyanins [16]. Anthocyanins are glycosylated compounds formed through glycosidic bonds between anthocyanidins and various monosaccharides, primarily glucose, arabinose, rhamnose, and galactose [17]. In some plant species, disaccharides or acylated sugars may also be involved [16,18]. Apple peels contain five major anthocyanins: cyanidin-3-galactoside, cyanidin-3-xyloside, cyanidin-3-rutinoside, cyanidin-3-arabinoside, and cyanidin-3-glucoside [19,20]. Among these, cyanidin-3-galactoside is the most abundant, accounting for more than 85% of the total anthocyanin content. The remainder includes cyanidin-3-arabinoside, cyanidin-3-glucoside, and other minor constituents [21]. Cyanidin-3-galactoside also predominates in the flesh of red-fleshed apples, though its proportion of total anthocyanins is relatively lower—for example, 39% in *Malus niedzwetzkyana* [22] and 68% in ‘Weirouge’ [23].

Anthocyanin biosynthesis is a branch of the flavonoid metabolic pathway, starting from phenylalanine and proceeding through a series of enzymatic reactions to form stable anthocyanins. Key enzymes involved include phenylalanine ammonia-lyase (PAL), cinnamate-4-hydroxylase (C4H), 4-coumaroyl-CoA ligase (4CL), chalcone synthase (CHS), chalcone isomerase (CHI), flavanone 3-hydroxylase (F3H), flavonoid 3′-hydroxylase (F3′H), flavonoid 3′5′-hydroxylase (F3′5′H), dihydroflavonol reductase (DFR), anthocyanidin synthase (ANS), and UDP-glycose: flavonoid glycosyltransferase (UFGT) [24,25,26,27]. Beyond biosynthetic enzymes, anthocyanin accumulation is also regulated by transcription factors, notably MYB, bHLH, and WD40-repeat proteins, which often form MYB-bHLH-WD40 (MBW) ternary complexes. These transcription factors bind to promoters of structural genes in the anthocyanin pathway, either activating or repressing their expression to modulate anthocyanin content [28,29]. MYB transcription factors constitute one of the largest transcription factor families in plants and play a pivotal role in regulating anthocyanin biosynthesis. They maintain the balance of anthocyanin accumulation across different plant tissues through positive or negative regulation of pathway genes [30]. In apple, several MYB transcription factors have been identified that regulate peel and flesh coloration, such as MdMYB1 and MdMYB3, which positively regulate anthocyanin accumulation in the fruit skin [31,32]. The MYB transcription factors MdMYB10 and MdMYB110a play crucial roles in the development of red flesh in apple fruit. MdMYB10 is an allele of MdMYB1 [33], and these two regulators are expressed during early and late fruit ripening stages, respectively [34]. In the red-fleshed cultivar ‘JPP35’, the expression levels of *MdMYB110a*, *CHS*, and *ANS* genes peaked approximately 40 days before harvest [35]. Similarly, in cultivars ‘Nakano Shinku’ and ‘Nakano no Kirameki’, the expression of *MdMYB110a* also reached its maximum level 30 days prior to harvest [36]. These findings can be applied in breeding programs for red-fleshed apples with enhanced nutraceutical value. Similarly, bHLH transcription factors possess DNA-binding and dimerization capabilities. For instance, the cold-induced bHLH transcription factor MdbHLH3 binds to the promoters of the anthocyanin biosynthetic genes *MdDFR* and *MdUFGT*, as well as the regulatory gene *MdMYB1*, activating their expression and thereby promoting anthocyanin accumulation and fruit coloration [37]. Another bHLH factor, MdILR3L, forms a complex with the MYB transcription factor MdCPCL. The MdCPCL–MdILR3L complex significantly enhances the transcription of downstream target genes *MdANS* and *MdUFGT*, further promoting anthocyanin synthesis [38]. WD40 proteins contain WD-repeat domains typically ending with aspartic acid (D) and tryptophan (W) residues. MdTTG1, the first WD40-repeat protein isolated in apple, interacts with MdbHLH3 to facilitate anthocyanin accumulation [39]. Additional transcription factors, such as MdWRKY10 [40] and MdNAC33 [41], are also involved in regulating anthocyanin biosynthesis in apple. Besides transcriptional regulation, apple peel coloration is influenced by environmental conditions, phytohormones, and cultivation practices [42,43,44].

Advances in molecular biology and omics technologies have enabled in-depth exploration of the anthocyanin biosynthetic pathway and its regulatory network in apple. This review systematically summarizes the structural characteristics, biosynthesis, molecular regulation, and environmental factors affecting anthocyanin accumulation in apples. We also propose future research directions to provide a theoretical foundation for improving fruit quality and developing functional apple varieties through breeding.

## 2. Methods

This review is based on articles retrieved from PubMed, Semantic Scholar, Web of Science, World Wide Science, and Embase using the following keywords: “apple anthocyanin”, “biosynthesis”, “MYB”, “bHLH”, “WD40”, “light”, “temperature”, “sugar”, “acid”, “auxin”, “ethylene”, “abscisic acid”, “jasmonic acid”, and “cytokinin”. In addition to a general search without year restrictions, a separate search was conducted for literature published between 2000 and 2025.

## 3. Pigment Types in Apple and Distribution Characteristics of Anthocyanins

### 3.1. Pigment Types Influencing Apple Color

The diverse coloration of apple peel is primarily attributed to the presence of anthocyanins. To date, five major anthocyanins have been identified: cyanidin-3-galactoside, cyanidin-3-glucoside, cyanidin-3-arabinoside, cyanidin-3-xyloside, and cyanidin-3-rutinoside [19,20]. Variations in the composition and relative abundance of these anthocyanins among different apple cultivars lead to a broad spectrum of peel colors, ranging from light pink and bright red to deep purple [45]. In addition to anthocyanins, apple peel color is also influenced by the relative proportions of chlorophyll and carotenoids [46]. Studies have shown that at similar anthocyanin levels, higher chlorophyll content results in a dull red hue, while lower chlorophyll content leads to a vivid red appearance [47,48,49,50]. For instance, the development of red color in ‘Royal Gala’ is not only due to anthocyanin accumulation during ripening but is also associated with significant chlorophyll degradation [51]. Carotenoids, a major group of terpenoid pigments, are responsible for the yellow coloration of apple peel and also contribute to fruit color development. In the cultivar ‘OPAL^®^’, an IAA-mediated degreening process occurs during maturation, wherein chlorophyll breakdown reveals underlying carotenoids, resulting in a yellow background color [51]. Yellow-skinned cultivars such as ‘Golden Delicious’ contain negligible anthocyanins; their yellow color is mainly due to carotenoids (e.g., β-carotene) and thorough chlorophyll degradation [52]. The continuous color spectrum from green to deep purple among different cultivars can be explained by differences in pigment composition and regulatory mechanisms, highlighting the synergistic role of multiple pigments in determining apple peel color.

### 3.2. Distribution Characteristics of Anthocyanins in Apple

Anthocyanins in apples are predominantly located in the peel, though they may also occur in the flesh, particularly in red-fleshed varieties [3,53,54]. The extent of pigmentation in the peel and flesh varies considerably among cultivars. Red-skinned cultivars such as ‘Fuji’, ‘Royal Gala’, ‘Gala’, and ‘Jonagold’ accumulate substantial anthocyanins in the peel [55,56,57]. Red-fleshed cultivars—including ‘RS-1’ (Red Moon Companie, Milano, Italy), ‘107/06’, ‘117/06’, ‘119/06’ (Lubera AG, Stein am Rhein, Switzerland), ‘Pink Pearl’, ‘Enbu’, ‘HFF33’, ‘HFF60’, ‘Kurenainoyume’, ‘Moonrouge’, and ‘Nakano no Kirameki’—contain anthocyanins in both the peel and flesh [58,59]. Anthocyanin biosynthesis occurs in the endoplasmic reticulum, and the pigments are subsequently transported and stored in the vacuoles of various cells and tissues [60]. In red-fleshed apples (e.g., Redlove^®^, Red Moon^®^, Kissabel^®^, and Lucy^TM^), anthocyanins accumulate mainly in the hypodermal layers and near vascular bundles [58,61]. In contrast, green- or yellow-skinned cultivars such as ‘Golden Delicious’ and ‘Granny Smith’ contain very low or negligible amounts of anthocyanins in the peel [51,62,63,64].

## 4. Basic Structure, Biosynthesis, and Transport of Anthocyanins

### 4.1. Basic Structure

**Figure 1:**
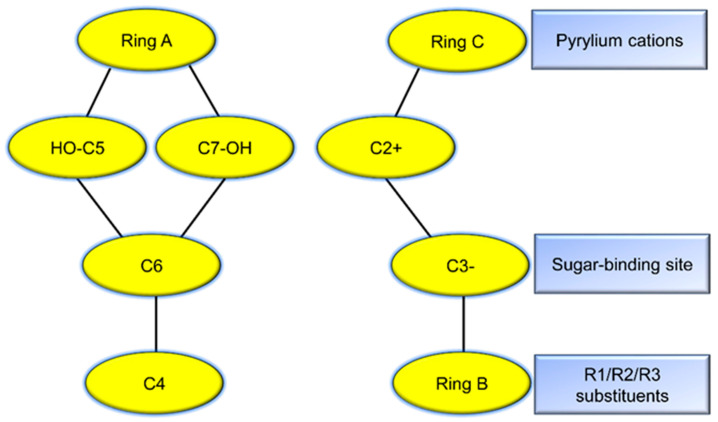
The basic structure of anthocyanins.

Anthocyanins are water-soluble pigments characterized by a C6–C3–C6 carbon skeleton (based on 3,5,7-trihydroxy-2-phenylbenzopyrylium), which consists of two aromatic rings (A and B) linked by an oxygen-containing heterocycle (C ring, benzopyrylium). As derivatives of flavonoids, anthocyanins share this common structural framework. The A ring (C6 structure) is typically substituted with hydroxyl groups (-OH) at the C5 and C7 positions. The C ring is a benzopyrylium moiety carrying a positive charge at the C2 position (flavylium cation), while the C3 position may be hydroxylated or glycosylated (forming anthocyanins). The B ring (C6–C3 structure) varies in its substitution patterns (-OH or -OCH_3_), and these modifications determine the specific type of anthocyanin (Figure 1) [65].

Based on the substitution patterns on the B ring, more than 20 distinct anthocyanidins have been identified to date [6]. Among these, six are widely prevalent in horticultural plants: cyanidin (Cy), pelargonidin (Pg), delphinidin (Dp), peonidin (Pn), malvidin (Mv), and petunidin (Pt) (Table 1) [14,66]. Each anthocyanin confers unique coloration: cyanidin derivatives typically appear red, pelargonidin derivatives orange-red, and peonidin derivatives magenta, while delphinidin, malvidin, and petunidin derivatives often exhibit blue-purple hues (Table 1) [67]. An increase in hydroxyl groups (e.g., delphinidin, with three -OH groups on the B ring) enhances bluish tones, whereas methoxyl group substitutions (e.g., malvidin, with two -OCH_3_ groups on the B ring) improve stability and shift color toward magenta [6]. In nature, anthocyanidins predominantly exist in the form of anthocyanin conjugated with monosaccharides (e.g., glucose, galactose) or disaccharides via glycosidic bonds, most commonly at the C3 position, and occasionally at C5 or C7 [17].

### 4.2. Biosynthesis

**Figure 2:**
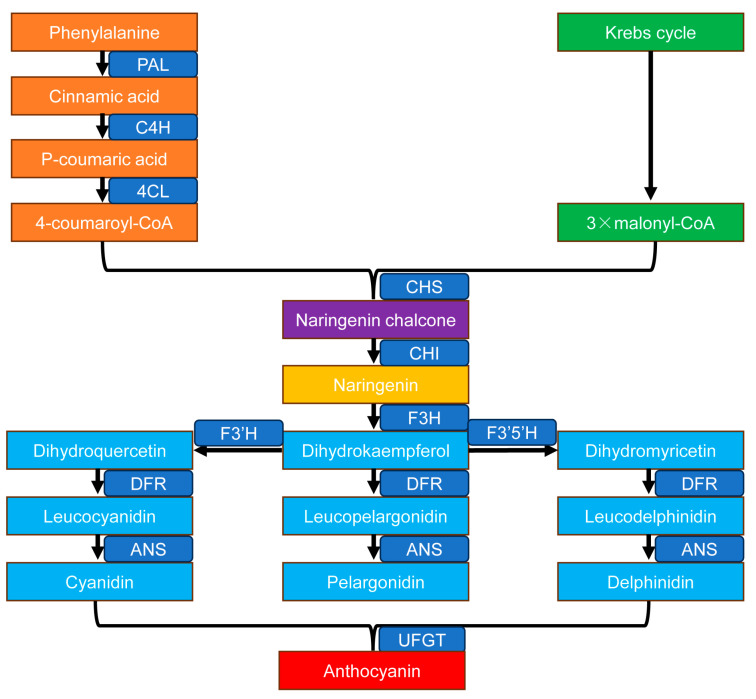
The anthocyanin biosynthetic pathway. The pathway begins with the conversion of phenylalanine to cinnamic acid catalyzed by phenylalanine ammonia-lyase (PAL). Cinnamate-4-hydroxylase (C4H) then combines with hydroxylates cinnamic acid to form p-coumaric acid, which is subsequently activated to 4-coumaroyl-CoA by 4-coumaroyl-CoA ligase (4CL). One molecule of 4-coumaroyl-CoA and three molecules of malonyl-CoA (from the Krebs cycle) are condensed by chalcone synthase (CHS) to produce naringenin chalcone. This chalcone is isomerized by chalcone isomerase (CHI) to form naringenin, the core flavanone structure. Naringenin is hydroxylated by flavanone 3-hydroxylase (F3H) to form dihydrokaempferol (DHK). From this point, the pathway diverges into three branches: DHK can be hydroxylated by flavonoid 3′-hydroxylase (F3′H) to yield dihydroquercetin (DHQ), which is then reduced by dihydroflavonol 4-reductase (DFR) to leucocyanidin, and finally oxidized by anthocyanidin synthase (ANS) to form cyanidin. Alternatively, DHK is directly reduced by DFR to leucopelargonidin and oxidized by ANS to yield pelargonidin. In a third branch, DHK is hydroxylated by flavonoid 3′,5′-hydroxylase (F3′5′H) to form dihydromyricetin (DHM), which is reduced by DFR to leucodelphinidin and oxidized by ANS to produce delphinidin. The unstable anthocyanidins (cyanidin, pelargonidin, delphinidin) are glycosylated by UDP-glucose: flavonoid glucosyltransferase (UFGT) to form stable anthocyanins, which are the final colored pigments.

Anthocyanin biosynthesis is a branch of the flavonoid metabolic network, initiating from phenylalanine and proceeding through a series of enzymatic reactions to form stable anthocyanins (Figure 2). This pathway can be divided into three main stages [24].

The first stage involves the general pathway leading to the formation of the flavanone skeleton. Phenylalanine is deaminated to cinnamic acid by PAL. Cinnamic acid is then converted to 4-coumaroyl-CoA by the actions of C4H and 4CL [24,68]. Subsequently, one molecule of 4-coumaroyl-CoA and three molecules of malonyl-CoA are condensed by CHS to form chalcone [9]. Chalcone is stereospecifically isomerized by CHI to naringenin, yielding the flavanone backbone [24,69].

The second stage comprises the branch pathway leading to anthocyanidin formation. Naringenin is hydroxylated by F3H to form dihydrokaempferol (DHK). DHK is further hydroxylated by F3′H and F3′5′H to produce dihydroquercetin (DHQ) and dihydromyricetin (DHM), respectively. DHK, DHQ, and DHM are then reduced by DFR to yield leucoanthocyanidins. Leucoanthocyanidins are oxidized by ANS to form colored anthocyanidins [24,70]. The substrate specificity of DFR is a key determinant of anthocyanidin diversity, with the three branches (DHK, DHQ, and DHM) leading to the formation of pelargonidin, cyanidin, and delphinidin, respectively [24,70].

The third stage involves modification reactions that generate diverse anthocyanins. UFGT is a key rate-limiting enzyme for anthocyanin accumulation, and its activity directly determines anthocyanin stability. Anthocyanidins are glycosylated by UFGT to form stable anthocyanins [24].

The expression patterns and functional roles of structural genes in the anthocyanin biosynthetic pathway have been extensively studied in apple. PAL, the rate-limiting enzyme in the first step of the pathway, exhibits two peaks in activity—during the early fruit stage and the maturation stage. Anthocyanin accumulation in young fruits is correlated with PAL activity, whereas no such correlation is observed during fruit maturation [71]. CHS, which constructs the basic flavonoid C-skeleton, is a key enzyme in the flavonoid pathway. In apple fruits, red cultivars such as ‘Jonagold’ and ‘Fuji’ show significantly higher expression levels of *CHS* and other structural genes compared to the non-red cultivar ‘Ohrin’ [72]. *CHS* expression remains high throughout both the young and mature fruit stages; however, it does not appear to regulate light-induced anthocyanin synthesis [73]. During fruit coloration in the red cultivar ‘Splendour’, anthocyanin content is significantly correlated with the expression of *CHI* [74]. F3H catalyzes the conversion of naringenin to dihydroflavonols, supplying essential precursors for anthocyanin biosynthesis. Its expression is markedly higher in red-skinned apples during coloration, indicating a crucial role in peel pigmentation [31]. DFR, utilizing NADPH as a cofactor, reduces dihydroflavonols such as dihydrokaempferol, dihydroquercetin, and dihydromyricetin to leucoanthocyanidins [75]. Consistent with this, *DFR* expression is significantly elevated in red apple varieties compared to non-red ones [31]. ANS, a key iron-dependent dioxygenase in the late stage of the pathway, catalyzes the conversion of leucoanthocyanidins to colored anthocyanidins [75]. In apples, red cultivars show high transcript abundance of *ANS* in the fruit skin, whereas it is nearly undetectable in non-red cultivars [76]. UFGT catalyzes the final step of anthocyanin biosynthesis, stabilizing anthocyanidins via glycosylation. Although *UFGT* expression varies significantly across fruit developmental stages, its transcript levels are closely associated with anthocyanin accumulation during fruit development in apple [77].

### 4.3. Transport of Anthocyanins

Anthocyanins are water-soluble pigments synthesized in the cytoplasm. They are soluble in water and alcohols but insoluble in organic solvents such as ether and chloroform. In nature, free anthocyanidins are rare; they are typically glycosylated with sugars such as glucose, galactose, arabinose, xylose, or rhamnose via glycosidic bonds to form stable anthocyanins [17]. These compounds are subsequently transported and accumulated in the vacuoles of plant cells [78]. Therefore, vacuolar membrane transporters play a crucial role in the storage and accumulation of anthocyanins. Three primary transport mechanisms facilitate the movement of anthocyanins from the cytoplasm into the vacuole: glutathione S-transferase (GST)-mediated transport, membrane transporter-mediated transport, and vesicle-mediated trafficking [79].

Putative anthocyanin transporters include proteins from the ATP-binding cassette (ABC) superfamily, particularly multidrug resistance-associated proteins (MRPs), as well as members of the multidrug and toxic compound extrusion (MATE) family [80]. In apple, the expression of *MdABCI17*, a tonoplast-localized ABC transporter, is positively correlated with anthocyanin accumulation during fruit ripening. Moreover, upregulation of *MdABCI17* in apple fruit and calli leads to significant induction of key anthocyanin biosynthetic genes, including *MdANS*, *MdCHS*, *MdCHI*, *MdDFR*, and *MdUFGT*, indicating a positive regulatory role of MdABCI17 in anthocyanin accumulation [80]. Glutathione S-transferases (GSTs) are also implicated in anthocyanin vacuolar sequestration [81]. For instance, MdGSTF6 encodes a critical GST transporter in apple fruit, whose expression is activated by MdMYB1 [82]. Functional characterization in transgenic apple calli demonstrated that the *MdGSTU12* gene positively regulates anthocyanin accumulation [83]. Additionally, MATE transporters MdMATE1/2 contribute to anthocyanin accumulation in the vacuole [82,84].

## 5. Molecular Regulatory Mechanisms of Anthocyanin Biosynthesis

The biosynthesis of anthocyanins is governed by a multi-tiered transcriptional regulatory network, primarily involving MYB, bHLH, and WD40 transcription factors. These often form MBW (MYB–bHLH–WD40) complexes that bind to cis-acting elements in the promoters of structural genes, thereby spatiotemporally regulating their expression [85,86].

### 5.1. MYB Transcription Factors

MYB transcription factors represent one of the largest transcription factor families in plants and play a central role in regulating anthocyanin biosynthesis in apples. Based on the number of MYB domain repeats, they are classified into four subfamilies: R1-MYB, R2R3-MYB, R3-MYB, and R4-MYB. Among these, R2R3-MYB proteins are the major regulators of anthocyanin synthesis [30].

Several MYB transcription factors involved in anthocyanin regulation have been identified in apple (Table 2), and they can be functionally categorized as either activators or repressors. For example, light-induced MdMYB1 activates the expression of *DFR* and *UFGT*, promoting anthocyanin accumulation in the fruit skin [31]. MdMYB10 and MdMYBPA1 are key regulators strongly associated with anthocyanin levels in red-fleshed apples [3,53] (Table 2). Knowledge of these regulatory mechanisms has been applied to improve apple quality. Molecular markers (e.g., allelic variations in MdMYB10) are used to accelerate the breeding of red-skinned cultivars [3,31], and transgenic overexpression of key regulators such as *MdMYB10* has successfully enhanced anthocyanin content [3].

Other activators include MdMYB3, which promotes anthocyanin accumulation by activating *DFR* and *UFGT* [32], and MdMYB9/11, which positively regulates anthocyanin and proanthocyanidin accumulation, also participate in jasmonate-induced pigment synthesis [87]. In contrast, several MYB repressors fine-tune anthocyanin biosynthesis. MdMYB16 suppresses anthocyanin synthesis via its C-terminal EAR repression domain by forming homodimers [88], while MdMYB6 indirectly inhibits anthocyanin production by reducing UDP-glucose and UDP-galactose levels [89].

MdMYB90-like directly activates anthocyanin biosynthetic genes (*MdCHS*, *MdCHI*, *MdANS*, *MdUFGT*) and indirectly enhances biosynthesis by upregulating other regulatory factors, such as MdbHLH3 and MdMYB1 [90]. Conversely, MdMYB306L activates the expression of the repressor *MdMYB17* and directly suppresses *MdDFR* by binding to its promoter [91]. Overexpression of *MdMYB24L* in ‘Orin’ apple calli elevated anthocyanin content and upregulated *MdUFGT* and *MdDFR* expression. Further analysis confirmed that MdMYB24L binds to MYB-binding sites in the promoters of these genes to activate their transcription [92]. Finally, the R3-MYB transcription factor MdCPCL positively regulates both ascorbate and anthocyanin levels; its overexpression enhances pigment accumulation, while knockdown reduces it [38].

**Table 2:** MYB transcription factors involved in anthocyanin biosynthesis in apples.

Gene Name	Position of Action	Positive/Negative Regulation	References
MdMYB1	Apple skin	Positive	[31]
MdMYB3	Apple skin	Positive	[32]
MdMYB6	Apple flesh	Negative	[89]
MdMYB9	Apple flesh	Positive	[87,93]
MdMYB10	Apple flesh	Positive	[3]
MdMYB11	Apple flesh	Positive	[87]
MdMYB12	Apple flesh	Positive	[94]
MdMYB16	Apple skin	Negative	[88]
MdMYB17	Apple peel and flesh	Negative	[95]
MdMYB24L	Apple flesh	Positive	[92]
MdMYB66	Apple skin	Positive	[96]
MdMYB88	Apple leaves	Positive	[97]
MdMYB90L	Apple skin	Positive	[90]
MdMYB110a	Apple skin	Positive	[98]
MdMYB111	Apple flesh	Negative	[99,100]
MdMYB114	Apple skin and flesh	Positive	[101]
MdMYB124	Apple leaves	Positive	[97]
MdMYB305	Apple flesh	Negative	[102]
MdMYB306L	Apple skin and flesh	Negative	[91]
MdMYB308L	Apple flesh	Positive	[103]
MdMYBL2	Apple flesh	Negative	[104]
MdMYBPA1	Apple flesh	Positive	[53,93]
MdMYBA	Apple skin	Positive	[54]
MdCPCL	Apple skin and flesh	Positive	[38]
MdMYB1R1	Apple flesh	Positive	[105]

### 5.2. bHLH Transcription Factors

bHLH (basic helix–loop–helix) transcription factors represent the second largest family of TFs in plants, characterized by a conserved bHLH domain that facilitates protein–protein interactions, notably with MYB proteins to form regulatory complexes [106]. In apple, MdbHLH3 is induced by low temperatures and interacts with MdMYB1 to activate anthocyanin synthesis [37]. MdbHLH33 binds to low-temperature response elements in the *MdMYBPA1* promoter, promoting the conversion of proanthocyanidins to anthocyanins [53]. Overexpression of *MdbHLH51* enhances anthocyanin production in the fruit skin of apple and in calli of ‘Gala’, indicating its regulatory role in pigmentation [107]. The bHLH TF MdPIF1 (PHYTOCHROME-INTERACTING FACTOR 1) positively regulates anthocyanin biosynthesis in both apple calli and fruits by directly binding to G-box elements in the promoters of *MdPAL* and *MdF3H*, thereby enhancing their transcription [108]. Conversely, MdbHLH162 acts as a negative regulator by interacting with MdbHLH3 and MdbHLH33, disrupting the formation of active MdMYB1-MdbHLH3/33 complexes and attenuating the transcription of *MdDFR* and *MdUF3GT* [42]. The R3-MYB TF MdCPCL interacts with the bHLH factor MdILR3L to form a complex that strongly activates the transcription of *MdANS*, promoting anthocyanin synthesis [38]. Additionally, MdMYB306L interacts with both MdbHLH33 and MdMYB17 to enhance their regulatory activities, facilitating anthocyanin biosynthesis [91]. MdMYB24L positively regulates the transcription of *MdDFR* and *MdUFGT* by binding to MYB-binding motifs in their promoters, and its interaction with the bHLH factor MdMYC2 further enhances *MdUFGT* transcription [92].

### 5.3. WD40 Repeat Proteins

WD40 proteins contain one or more conserved WD40 domains, each comprising approximately 40 amino acid residues with characteristic GH and WD dipeptides at the N- and C-termini, respectively [109,110]. The most studied WD40 protein in plant anthocyanin biosynthesis is TRANSPARENT TESTA GLABRA1 (TTG1). WD40 proteins, such as MdTTG1, do not bind DNA directly but form stable MBW (MYB–bHLH–WD40) complexes essential for regulating the spatial and temporal expression of anthocyanin structural genes [85,86,111]. The WD40 protein CONSTITUTIVELY PHOTOMORPHOGENIC 1 (COP1) has been identified in *Arabidopsis thaliana*, *Oryza sativa*, and *Malus* × *domestica* [112]. In apple, MdCIP1 (MdCOP1-INTERACTING PROTEIN 1) interacts with MdCOP1 via its coiled-coil domain. *MdCIP1* overexpression in *cop1-4* mutants results in photomorphogenic phenotypes similar to *cop1-4*, suggesting that COP1 acts epistatically to CIP1. Transient assays indicate that MdCIP1 suppresses anthocyanin biosynthesis through the MdCOP1 pathway [113]. MdTTG1 promotes anthocyanin accumulation by interacting with the bHLH TF MdbHLH3 (which itself interacts with MYB proteins) but does not interact directly with MYB proteins [39]. Another WD40 protein, MdTT8, a homolog of Arabidopsis AtTT8, participates in MBW complex formation [111].

### 5.4. Other Transcription Factors Affecting Apple Fruit Coloration

Beyond MYB and bHLH factors, several other TFs influence anthocyanin synthesis, often by binding to the promoters of MYB genes. These include the bZIP TF MdHY5 [114], MdWRKY11 [115], and MdBBX20 (a B-box zinc finger protein), which binds to the *MdMYB1* promoter to promote anthocyanin accumulation under UV radiation and low temperatures [116]. MdbZIP4 upregulates *MdMYB114* expression to enhance anthocyanin biosynthesis [101]. The ethylene response factor MdERF1B significantly increases anthocyanin and proanthocyanidin (PA) content in overexpressing calli by activating the promoters of *MdMYB9* and *MdMYB11* [111,117]. The NAC TF MdNAC52 promotes anthocyanin and PA biosynthesis by binding to the promoters of *MdMYB9* and *MdMYB11* [118]. MdMYB111 and MdWRKY40 form homodimers that bind to the W-box in the *MdANS* promoter, alleviating the repression by MdMYB111 and promoting anthocyanin synthesis [100]. MdWRKY71 interacts with MdMADS1 to activate its transcription and enhances the transcriptional activation of *MdCHS* and *MdUFGT* by MdMADS1. Additionally, MdWRKY71 independently binds to and activates the promoters of *MdANS* and *MdDFR*, revealing a cooperative role in regulating anthocyanin biosynthesis [105]. The bZIP TF MdHY5, induced by light and ABA treatment, promotes anthocyanin accumulation in apple calli by regulating *MdMYB10* and downstream biosynthetic genes [114].

## 6. Other Factors Influencing Anthocyanin Accumulation in Apple

Anthocyanin biosynthesis is influenced by various environmental and internal cues, including light, temperature, phytohormones, and sugar/acid balance, which integrate into the regulatory network to affect apple fruit coloration [44,102,119,120,121].

### 6.1. Light

**Figure 3:**
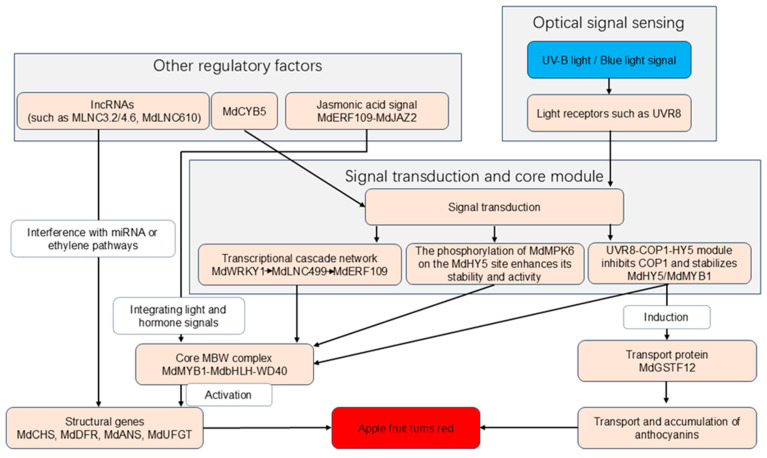
Molecular mechanism of light-regulated anthocyanin biosynthesis in apple.

Light is the most critical environmental factor regulating anthocyanin accumulation (Figure 3). Bagging inhibits anthocyanin synthesis, while debagging and light exposure rapidly induce pigment accumulation [122]. Reflective films and supplemental lighting can enhance anthocyanin content [119,123]. UV-B and blue light are most effective in promoting anthocyanin synthesis, whereas far-red light is inhibitory [124]. UV-B acts through the UVR8-COP1-HY5 signaling module to induce anthocyanin-related gene expression [125]. Light promotes the expression of structural genes and TFs, whereas their expression is suppressed in darkness [72,126]. Light perception activates photoreceptors and downstream signaling cascades [127]. For instance, light induces *MdMYB1* expression [31], but in the dark, MdCOP1 ubiquitinates and degrades MdMYB1; light inhibits nuclear translocation of MdCOP1, stabilizing MdMYB1 [128]. Extended photoperiods using LED lighting after sunset enhance anthocyanin synthesis in the skin and sugar accumulation in the flesh, mediated by MdWRKY40 (which binds to the MdGSTF12 promoter) and MdMYB108, respectively [129]. Two lncRNAs, MLNC3.2 and MLNC4.6, act as decoys for miR156a, preventing the cleavage of SPL2-like and SPL33 transcripts, thereby promoting light-induced anthocyanin biosynthesis [130]. Phosphorylation of MdHY5 by MdMPK6 under light prevents its ubiquitination by MdCOP1, leading to increased anthocyanin gene expression and accumulation [131]. MdGST12, regulated by MdWRKY26 and MdHY5, forms homodimers and interacts with MdUFGT and MdDFR under light to facilitate anthocyanin accumulation [132]. A light-induced transcriptional cascade involving MdWRKY1, MdLNC499, and MdERF109 promotes anthocyanin biosynthesis [133]. High-intensity light promotes anthocyanin accumulation more effectively than moderate light by inducing ethylene biosynthesis and activating anthocyanin genes; the lncRNA MdLNC610, induced by strong light, regulates *MdACO1* expression to enhance anthocyanin accumulation and ripening [134]. *MdCYB5*, a cytochrome b5 gene responsive to light, promotes anthocyanin accumulation and coloration when overexpressed [135].

Light Perception: Photoreceptors (e.g., UVR8) perceive UV-B/blue light signals. Signal Transduction: UVR8-COP1-HY5 module: UV-B inhibits MdCOP1, preventing ubiquitination-mediated degradation of MdHY5 and MdMYB1. Phosphorylation regulation: Light-activated MdMPK6 phosphorylates MdHY5, enhancing its stability and transcriptional activity. Transcriptional cascades: Light induces transcription factors (e.g., MdWRKY1), which activate lncRNAs (e.g., MdLNC499) and downstream regulators (e.g., MdERF109), further amplifying the signal. MBW Complex Activation: Stabilized MdMYB1 forms a complex with MdbHLH and WD40 proteins, activating structural genes in the anthocyanin pathway (e.g., *MdCHS*, *MdDFR*, *MdANS*, *MdUFGT*). Transport and Accumulation: Light-induced MdGSTF12 facilitates anthocyanin transport into the vacuole. Additional Regulatory Factors: lncRNAs (e.g., MLNC3.2, MdLNC610), MdCYB5, and jasmonic acid signaling components (e.g., MdERF109-MdJAZ2) integrate light and hormonal signals to fine-tune anthocyanin synthesis.

### 6.2. Temperature

**Figure 4:**
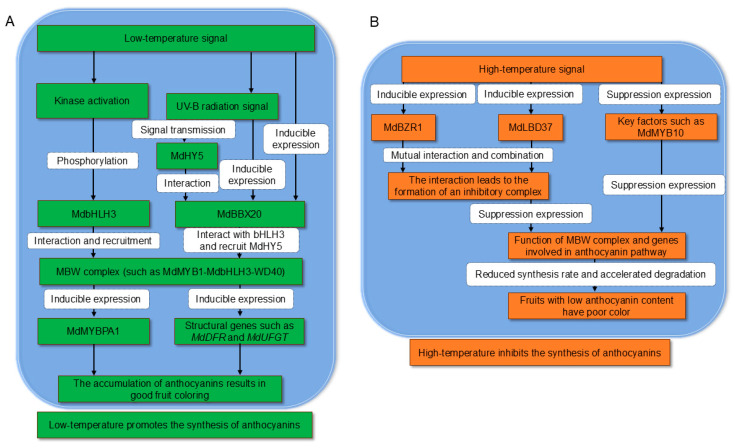
Temperature Regulation of Anthocyanin Biosynthesis via MYB-bHLH-WD40 Complex in Apple. (**A**) Low temperature promotion pathway: Low temperature and UV-B radiation induce phosphorylation of MdbHLH3, enhance *MdBBX20* expression, and promote MdHY5-MdBBX20 interaction. These signals converge to enhance the activity of MBW complex (e.g., MdMYB1-MdbHLH3-WD40), which activates structural genes (*MdDFR*, *MdUFGT*, *CHS*, *ANS*) and transcription factors (MdMYBPA1). MdbHLH33 binds to the Low-Temperature Response (LTR) element in *MdMYBPA1* promoter, shifting biosynthesis from proanthocyanins to anthocyanins. Concurrently, MdMYB23 activates *MdCBF1/2* and *MdANR*, enhancing proanthocyanin biosynthesis and cold tolerance. (**B**) High temperature suppression pathway: High temperature induces expression of repressors *MdBZR1* and *MdLBD37*, which form a positive feedback loop to reinforce each other’s expression. This repressor complex inhibits anthocyanin pathway genes and potentially disrupts MBW complex stability. High temperature also suppresses key activators (MdMYB10) and accelerates anthocyanin degradation, leading to reduced pigmentation.

Temperature significantly affects anthocyanin synthesis (Figure 4). Fruits grown in vinyl houses (heat-treated) showed poor coloration and lower gene expression and pigment accumulation compared to those under irrigation (cooled) or ambient conditions [120]. Moderate low temperatures combined with light can maintain or enhance postharvest anthocyanin content [119]. Low temperature induces phosphorylation of MdbHLH3, enhancing its interaction with MdMYB1 and activating the anthocyanin pathway [119]. MdbHLH33 binds to low-temperature response elements (LTRs) in the *MdMYBPA1* promoter to promote anthocyanin accumulation under cold conditions [53]. ROS1 promotes anthocyanin accumulation under low-temperature conditions in apple by demethylating the promoters of the anthocyanin-related genes *MdF3′H* and *MdUFGT* [136]. High temperatures reduce anthocyanin synthesis and accelerate degradation by suppressing key regulators like MdMYB10 [137] and potentially destabilizing MBW complexes [99]. MdMYB23 enhances cold tolerance by activating *MdCBF1*, *MdCBF2*, and *MdANR*, promoting PA biosynthesis [138]. Low temperature and UV-B radiation synergistically increase the expression of *CHS*, *ANS*, and *UFGT*, enhancing anthocyanin accumulation [119]. MdBBX20, in concert with MdbHLH3 and MdHY5, integrates low-temperature and UV-B signaling to promote anthocyanin synthesis [116]. High temperatures induce MdBZR1, which suppresses anthocyanin biosynthesis by forming a positive feedback loop with MdLBD37 [139]. High temperatures also inhibit *MdMYB10* expression, reducing anthocyanin synthesis and impairing coloration [137].

### 6.3. Phytohormones

Auxin influences anthocyanin accumulation via ARF-Aux/IAA interactions. MdARF13 suppresses anthocyanin biosynthesis by binding to the *MdDFR* promoter, and overexpression of *MdIAA121* attenuates this repression [99].

Ethylene, a key hormone in fruit ripening, promotes anthocyanin synthesis [140]. The accumulation of anthocyanins in the peel of ‘Royal Gala’ apples is likely regulated ethylene-dependently, through the mediation of MdMYB1, which links ethylene signaling to anthocyanin biosynthesis and vacuolar transport [51]. Ethylene levels increase during ripening alongside anthocyanin accumulation. The ethylene response factor MdERF1B binds to the promoters of *MdMYB9*/*11* to promote anthocyanin and PA accumulation [117]. Ethylene treatment induces coloration and *MdMYB1* expression; MdMYB1, in turn, positively regulates ethylene biosynthesis by interacting with the *MdERF3* promoter [141]. In apple, the E3 ubiquitin ligases SINA4 and SINA11 regulate anthocyanin biosynthesis by targeting the IAA29-ARF5-1-ERF3 module, thereby mediating crosstalk between auxin and ethylene signaling pathways [142]. Additionally, MdERF1B, a central responder to jasmonate (JA) and ethylene signals, acts as a positive regulator of anthocyanin synthesis. MdEIL1 directly binds to the promoter of *MdERF1B* and upregulates its expression, thereby promoting anthocyanin accumulation [143].

Abscisic acid (ABA) coordinates carbon and nitrogen metabolism in late fruit development, reducing nitrogen and increasing carbon/sugar accumulation, providing substrates for anthocyanin synthesis. ABA activates MdbZIP44, which interacts with MdMYB1 to enhance downstream gene expression [144]. The NAC TF MdNAC77L is highly correlated with anthocyanin levels; its overexpression increases anthocyanin content in apple and strawberry by directly binding to the promoters of *DFR*, *ANS*, and *UFGT*; ABA strongly induces *MdNAC77L* expression, activating these target genes [145].

Methyl jasmonate (MeJA) rapidly degrades MdJAZ2, releasing MdbHLH3 to promote anthocyanin synthesis [87]. MdNAC72 and MdABI5 form a cascade that positively regulates anthocyanin biosynthesis. MdJAZ2 disrupts MdNAC72-MdABI5 interaction, and GA repressor MdRGL2a sequesters MdJAZ2, releasing MdNAC72. The E3 ligase MdSINA2 promotes MdNAC72 degradation in response to JA and GA signals [146]. MdZFP7 promotes anthocyanin synthesis by activating MdMYB1; MdJAZ2 interferes with MdZFP7 function, and MdRGL3a counteracts this by sequestering MdJAZ2. The E3 ligase MdBRG3, antagonistically regulated by JA and GA, promotes MdZFP7 degradation [147]. GA repressor MdRGL2a antagonizes the anthocyanin repressor MdbHLH162 by sequestering it from MdbHLH3/33 complexes. JA repressors MdJAZ1/2 interfere with MdRGL2a-MdbHLH162 interaction, enabling MdbHLH162 to integrate GA and JA signals to negatively regulate anthocyanin biosynthesis [42].

Cytokinin (CTK) induces anthocyanin biosynthesis in a sugar-dependent manner linked to the redox state of photosynthetic electron transport [148]. CTK induces anthocyanin accumulation in apple calli [144]. The WOX TF MdWOX8 enhances anthocyanin biosynthesis by activating *MdHY5* expression, which serves as an integrator of strigolactone (SL) and GA signaling. However, the SL repressor MdSMXL8 inhibits this activation of the *MdHY5* promoter by MdWOX8. In contrast, the GA repressor MdRGL3a not only enhances MdWOX8’s activity but also disrupts its interaction with MdSMXL8. The E3 ligase MdSINA1 targets MdWOX8 for ubiquitination and degradation in response to SL and GA signals [149].

### 6.4. Sugars and Acids

Sugars serve as both precursors and signaling molecules for anthocyanin biosynthesis [150]. In the cytoplasm, unstable anthocyanidins are glycosylated by UFGT to form stable anthocyanins [25,151]. Genes promoting soluble sugar accumulation are often linked to anthocyanin accumulation. High sugar levels suppress the U-box E3 ubiquitin ligase MdPUB29, which ubiquitinates MdbHLH3, affecting ethylene biosynthesis and negatively regulating anthocyanin accumulation [152]. Sucrose is the most effective sugar in promoting anthocyanin accumulation, inducing the expression of *AtPAP1* and *AtDFR* in Arabidopsis [153]. In apple, sucrose induces *MdMYB75/PAP1* expression to promote anthocyanin synthesis [153]. Sucrose transporters (SUCs) may act as receptors in sucrose-induced anthocyanin synthesis; suc1 mutants show inhibited anthocyanin accumulation on media containing 3% sucrose [154,155]. UDP-galactose is a major substrate for anthocyanin synthesis in apple [156]. MdSUT4 transports sucrose from the vacuole to the cytoplasm, increasing precursor availability for anthocyanin synthesis [157]. Conversely, MdMYB6 suppresses anthocyanin synthesis by regulating the monosaccharide transporter MdTMT1, reducing UDP-glucose and UDP-galactose levels [89]. Glucose activates the hexokinase MdHXK1, which phosphorylates MdbHLH3 to mediate fruit coloration [158].

The R2R3-MYB TF MdMYB305, identified in red-fleshed apple progeny, is positively correlated with sugar content but negatively correlated with anthocyanin levels. It binds to and activates sugar-related genes (*MdCWI1*, *MdVGT3*, *MdTMT2*) while repressing anthocyanin genes (*MdF3H*, *MdDFR*, *MdUFGT*). MdMYB305 competes with MdMYB10 for binding to MdbHLH33, balancing sugar and anthocyanin accumulation [102]. Glucose promotes anthocyanin accumulation in many plants. In apple, the glucose sensor MdHXK1 interacts with and phosphorylates MdbHLH3 at Ser361 under glucose stimulation. This phosphorylation stabilizes MdbHLH3 and enhances its transactivation of anthocyanin genes, increasing biosynthesis [158,159].

Research on organic acids’ role in anthocyanin regulation is limited, primarily focusing on pH-dependent stability. Anthocyanin color expression is also strongly influenced by vacuolar pH and co-pigmentation with flavonols. Anthocyanins exist in four forms (flavylium cation, quinoidal base, carbinol pseudobase, chalcone) that interconvert with pH, yielding different colors [121]. The flavylium cation (red) dominates at pH < 2; the carbinol pseudobase (colorless) at pH 4–6; the quinoidal base (purple) at pH 6–8; and the chalcone (colorless) at pH > 8. Higher pH generally accelerates anthocyanin degradation [121]. Malate is the primary organic acid in apples, with minor amounts of citrate, tartrate, and oxalate. Only citrate has been linked to anthocyanin synthesis [160].

## 7. Conclusions

Significant progress has been made in understanding apple anthocyanin biosynthesis over recent decades. Cyanidin-3-galactoside is the major pigment in apple skin, synthesized through a series of enzymatic steps in the phenylpropanoid and flavonoid pathways. The MBW transcriptional complex, particularly MYB TFs like MdMYB10 and MdMYB1, forms the core regulatory network. Environmental factors (light, temperature) and internal signals (hormones, sugars) are integrated into this network to modulate anthocyanin accumulation. These findings provide a theoretical basis for quality improvement and functional apple breeding. However, transport mechanisms remain less understood. Although MdGSTF6 (activated by MdMYB1) and MATE transporters MdMATE1/2 are involved in vacuolar sequestration, key knowledge gaps persist. These include the potential role of ABCC transporters (e.g., homologs of maize ZmMRP3) in glutathione-coupled transport, the mechanism of vesicle trafficking (e.g., analogous to Arabidopsis EXO70B1) in compartmentalization, the impact of modifications (e.g., acylation) on transporter specificity (e.g., MdMATEs), and the role of vacuolar stabilizers (e.g., grape VP24-like proteins).

Furthermore, epigenetic regulation represents the most understudied area in current research on apple anthocyanin biosynthesis. The accumulation of anthocyanins exhibits remarkable developmental stage specificity and environmental plasticity, strongly implying a central role for epigenetic mechanisms. Key questions remain unresolved: Does the methylation status of promoters of critical genes such as *MdMYB10* determine their expression variation across different cultivars and tissues—for instance, why flesh turns red while the peel does not? Are environmental signals, such as low temperature and high light, dynamically modulating chromatin accessibility of anthocyanin structural genes and transcription factors through mechanisms like demethylation? How do histone modifications—such as the activating mark H3K4me3, repressive mark H3K27me3, and acetylation—respond to light and temperature signals to precisely regulate the transcription of anthocyanin biosynthetic gene clusters (e.g., *CHS*, *DFR*, *ANS*)? Although this review mentions the involvement of lncRNAs (e.g., *MLNC3.2*, *MLNC4.6*) and miRNAs (e.g., *miR156a*), the nature of the competing endogenous RNA (ceRNA) network—incorporating miRNA–lncRNA–circRNA interactions—that finely tunes the expression of key transcription factors like *MdMYB* remains unclear. How do these non-coding RNAs integrate environmental cues and relay signals to the chromatin or post-transcriptional level?

Future studies should employ multi-omics approaches—including transcriptomics, proteomics, metabolomics, methylomics, and chromatin accessibility assays—to comprehensively identify key factors involved in anthocyanin transport and epigenetic regulation. CRISPR-based gene editing targets may include MYB family transcription factors such as *MdMYB10*, *MdMYB1*, *MdMYB16*, and *MdMYB6* (particularly repressors like *MdMYB16* and *MdMYB6*), as well as bHLH factors like *MdbHLH3* and *MdbHLH33*, which play pivotal roles in anthocyanin regulation. Additionally, suppressing negative regulators such as *MdBZR1*, *MdLBD37* (which inhibits anthocyanin under high temperature), *MdMYB306L*, and *MdbHLH162* via CRISPR could enhance anthocyanin accumulation. Genes involved in anthocyanin transport, including *MdGSTF6*, *MdMATE1/2*, and *MdABCI17*, are also potential editing targets to improve pigment compartmentalization.

Precise editing of these genes using CRISPR holds promise for developing apple varieties with higher anthocyanin content and more vivid coloration. Functional validation of candidate transporter genes and epigenetic regulators will be essential to elucidate their mechanisms. Deciphering the complete signaling cascade from environmental cues to epigenetic modifications, transcriptional regulation, and transport/storage will provide novel theoretical targets and molecular strategies for breeding apple cultivars with enhanced coloration and stress resistance.

## Figures and Tables

**Table 1:** Structural classification of common anthocyanidins based on B-ring substitutions.

Name	R_1_	R_2_	R_3_	Typical Color (Under Acidic Conditions)
Pelargonidin (Pg)	-H	-OH	-H	Orange-red
Cyanidin (Cy)	-OH	-OH	-H	Red
Delphinidin (Dp)	-OH	-OH	-OH	Blue-violet
Peonidin (Pn)	-OCH_3_	-OH	-H	Reddish-purple
Petunidin (Pt)	-OCH_3_	-OH	-OH	Deep-purple
Malvidin (Mv)	-OCH_3_	-OH	-OCH_3_	Bluish-purple

## Data Availability

No new data were created or analyzed in this study. Data sharing is not applicable to this article.
